# Supplementary material for: Content-rich biological network constructed by mining PubMed abstracts
Source: BMC Bioinformatics. 2004 Oct 8;5:147. doi: 10.1186/1471-2105-5-147 (PMC528731; doi:10.1186/1471-2105-5-147)
Supplement: Additional File 2 — The original results of the above study (non-essential files are deleted to keep the file size under the limit set by BMC bioinformatics). [file 1471-2105-5-147-S2.bz2 › chilibotAdditionalFile2/dip05/266ID8119416E1068/html/PPP1CC_PPP1R2.html]

 


 **PPP1CC** and **PPP1R2** 
  
Found 1 abstracts in PubMed, retrieved 1.  
 

 What does Google say? 
 PDF only 
| .edu only 

---

**Interactive relationship** (e.g. stimulation, inhibition, etc)

**Non-interactive relationship** (e.g. studied together, co-existance, homology, etc.)

- The PP1 genes examined were three catalytic subunit genes, PPP1CA, PPP1CB and  **PPP1CC** , and six regulatory subunit genes, PPP1R1A,  **PPP1R2** , PPP1R5, PPP1R6, PPP1R7 and PPP1R8.  Ref: 11251179 Int J Oncol, 2001
